# Inflation osteoplasty: in vitro evaluation of a new technique for reducing depressed intra-articular fractures of the tibial plateau and distal radius

**DOI:** 10.1007/s10195-012-0185-z

**Published:** 2012-03-06

**Authors:** Brandon Broome, Cyril Mauffrey, Jeremy Statton, Mike Voor, David Seligson

**Affiliations:** 1 Department of Orthopedic Surgery, University of Louisville, 210 E. Gray Street, Ste. 1003, Louisville, KY 40202 USA; 2 Orthopaedic Traumatology, Denver Health Medical Centre, 777 Bannock street, Denver, CO 80204 USA; 3 University of Louisville, Louisville, KY USA; 4 Orthopedic Bioengineering, University of Louisville, Louisville, KY USA; 5 Department of Orthopedics, University of Louisville, Louisville, KY USA

**Keywords:** Articular fractures, Fracture fixation, Knee injuries, Inflatable bone tamp

## Abstract

**Background:**

Anatomic reduction of intra-articular fractures of the tibial plateau and distal radius can be difficult to achieve. Treatment goals are centered on restoring the native anatomy and articular congruency. Several surgeons in the USA have begun using an inflatable bone tamp to reduce these fracture patterns. The concept is built on the success of the tamp in kyphoplasty of the spine, but it has yet to be tested in the lab for use in the extremities. We performed an investigation into the safety and efficacy of using an inflatable bone tamp for intra-articular fracture reduction of the tibial plateau and distal radius.

**Materials and methods:**

Paired cadaveric specimens were obtained for a total of six proximal tibias and six distal radii. Intra-articular depression-type fractures were created in all specimens. The inflatable bone tamp was then used to reduce the depression. For comparison, the tibias were fractured on the medial and lateral side and a conventional metal tamp was used on the contralateral side of the balloon. Fine-cut micro-computed tomography (CT) scans were performed on all intact specimens, which were then fractured, and again after fracture reduction. CT data was used to measure the amount of restoration of the normal anatomy and to compare the effectiveness of the balloon to conventional methods.

**Results:**

The inflatable bone tamp was equivalent to conventional methods in large, minimally displaced fracture fragments and proved superior when comminution was present at the articular surface. No instances of overreduction or penetration into the joint were encountered with the balloon, whereas this was a common occurrence with conventional metal tamps. The inflatable tamp was successful in reducing all distal radius fractures without complication.

**Conclusion:**

Anatomic reduction of impacted articular fractures should be the goal of any treating surgeon. In our cadaveric models, we have shown the inflatable bone tamp to be safe and effective in reducing depressed articular fractures around the tibial plateau and distal radius. The balloon offers the advantage of being minimally invasive and creating a symmetric, contained defect to hold bone filler for subchondral support.

## Introduction

Intra-articular fractures with joint congruity loss often carry a poor prognosis despite proper management [1–3]. Treatment complexity rises as the energy of the injury increases. Attempts to restore articular congruity must be combined with methods that protect soft tissues and preserve vascularity. Current techniques for managing depressed intra-articular fractures are similar regardless of the joint involved. Access to the subchondral bone is gained through a cortical window or other fenestration in the metaphyseal bone. A tamp or elevator is then inserted and used to elevate the depression en bloc. Bone graft or cancellous chips are often used to assist in elevating the fragments. The resulting void must then be filled with bone graft or bone-graft substitute to provide stability to the reduced fragment. These maneuvers may be imprecise and leave behind some degree of residual defect.

All intra-articular fractures share the common need to restore articular alignment and congruity. It is widely accepted that joint depression fractures with concomitant fractures of the surrounding metaphyseal struts must be treated operatively. In fact, several authors have recommended giving priority to restoring overall joint alignment and stability first, with less emphasis on restoring a completely smooth articular surface [1, 2, 4]. Good outcomes and low rates of posttraumatic arthritis have been reported for tibial plateau fractures with residual displacement ≥3 mm as long as the joint mechanical alignment and stability are restored [5, 6]. Much controversy remains regarding the treatment of isolated joint depression fractures. Most authors prefer minimal displacement of no more than 2–3 mm in order to consider nonoperative treatment. However, it has been reported that stepoffs upward of 10 mm can be tolerated in the proximal tibia [4]. When significant comminution is encountered, anatomic reduction may be impossible and some residual deformity must be accepted. Acceptable limits for joint surface displacement have yet to be agreed upon and vary depending on the joint involved. It seems intuitive, however, that a more precise reduction of the articular surface should result in a better outcome, as joint-surface congruency is restored. Spahn et al. [7] show that severe osteoarthritis involving joint congruity loss (grade IV lesion) was associated with significantly worse outcomes. Histological studies show that chondrocytes reproducibly undergo programmed cell death following intra-articular fracture [8, 9]. It should be inferred, then, that a more congruent articular surface will minimize later articular dysfunction. Perhaps the currently “acceptable” parameters regarding articular surface reduction are in place based on results with conventional techniques. These methods cannot always restore completely the native anatomy either due to fracture pattern or insufficient reduction tools/methods.

We sought to test the safety and efficacy of an inflatable bone tamp in performing intra-articular fracture reductions. The tamp consists of a balloon that can be inflated within the bone and subsequently removed. Much success has been documented with this technique in the spine for percutaneous kyphoplasty. Recent case reports document the success of using the inflatable tamp in the extremities. These reports include treating fractures of the calcaneus, cuboid, distal radius, tibial plateau, and acetabulum [10–13]. Despite preliminary success in these case reports, no laboratory study has been performed to document the safety or efficacy of this method.

It was our thought that with an inflatable device, a more congruent surface could be restored via a minimally invasive approach with less disruption of the subchondral bone. Our hypothesis was that the balloon would produce a better fracture reduction without joint penetration or fracture propagation. Even if the reduction proved to be equivocal to conventional methods, the balloon has the benefit of being less invasive and leaving behind a contained defect of known size that can then be filled with bone graft or other filler.

## Materials and methods

**Fig. 1:**
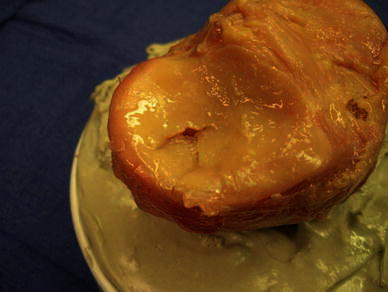
Depression fracture of the medial tibial plateau

Tibial plateau fractures were created in the Orthopaedic Bioengineering Laboratory using paired cadaveric specimens, a weight-drop method, and a spherical indenter. The proximal tibias were mounted in 10-cm-diameter polyvinylchloride (PVC) caps using a commercially available epoxy resin (Bondo, Dynatron Corp., Atlanta, GA, USA). A modified pipe clamp was tightened around the maximum circumference of the proximal tibia at the margin of the joint surface to prevent excessive split fractures during dynamic loading. A 2.54-cm-diameter steel ball was placed on the tibial condyle and a 5.5-cm-diameter pipe was held vertically above the ball. A 5-cm-diameter cylindrical weight weighing 11 N was dropped from a height of 33 cm onto the top of the ball. The resulting energy of the impact was approximately 3.6 Nm, and the impact velocity was approximately 2.5 m/s. This method reliably produced a depression-type plateau fracture within one or two drops of the weight (Fig. 1).

Each tibial plateau was fractured on both the medial and lateral sides. For comparison, all tibias underwent reduction of either the medial or lateral plateau with the balloon, and a conventional tamp was used on the contralateral side. For the conventional method, a small cortical window was made and a metal tamp was used to manually elevate the depression. With the balloon method, a working cannula was inserted parallel to the joint line in the metaphyseal bone. A trocar was passed to the midpoint of the tibia and withdrawn, leaving a path for balloon passage. The balloon was then inserted through the cannula and inflated, subsequently elevating the fracture. Ballon used was the Kyphon/Medtronic, Inc., Sunnyvale, CA, USA. This device is known as the Inflatable Bone Tamp (IBT). The IBT is designed to compress cancellous bone and/or move cortical bone as it inflates. It comprises three biocompatible parts: a proximal luer fitting, a central catheter, and a distal inflatable tip with radiopaque markers. Balloon inflation is achieved using an external inflation syringe filled with radiopaque dye. Fine-cut micro-computed tomography (CT) scans were performed on all intact specimens, following fracture, and again after being reduced. CT data ere used to quantify the fracture volume and displacement and the amount of restored normal anatomy. Data were used to judge restoration efficacy of the normal anatomy and to compare balloon efficacy to conventional methods. The same technique was used to create die-punch fractures of the distal radius. The height of the weight drop was altered to create different degrees of depression for comparison. Only the balloon technique was used to reduce distal radius fractures. The same CT scan protocol was used on all distal radius fractures to evaluate reduction efficacy.

## Results

**Fig. 2:**
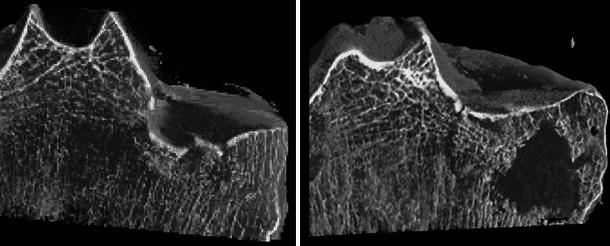
Comminuted depression reduced with the balloon technique

**Fig. 3:**
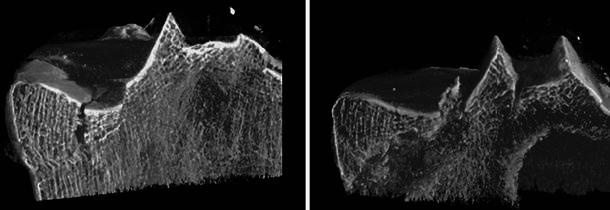
Comminuted depression with overreduction by a metal tamp

Six paired proximal tibias and six paired distal radii were used for the study. A total of 12 tibial plateau fractures were created (six medial and six lateral) and included in the study. One distal radius fracture was not suitable for use with either technique, leaving a total of five distal radius fractures for comparison. Results are shown in Table 1. In the presence of comminution at the articular depression, the balloon achieved superior results, with minimal residual deformity (Fig. 2). By comparison, the metal tamp was unable to adequately reduce articular comminution and easily penetrated the joint or overreduced the articular surface (Fig. 3).

**Table 1:** Tibial plateau results

	Plateau	Instrumentation	Fracture characteristics	Quality production
Tibial pair 1				
Right	Medial	Metal tamp	7 mm depression, comminution	Over reduction, penetration of joint surface
	Lateral	Balloon	4 mm depression	No/minimal residual defect
Left	Medial	Balloon	7 mm depression, comminution	No/minimal residual defect
	Lateral	Metal tamp	4 mm depression	Over reduction, cancellous bone into joint
Tibial pair 2				
Right	Medial	Metal tamp	Small depression <5mm	No/minimal residual defect
	Lateral	Balloon	Small depression <5mm	No/minimal residual defect
Left	Medial	Balloon	Small depression <5mm	No/minimal residual defect
	Lateral	Metal tamp	Small depression <5mm	1 mm final stepoff
Tibial pair 3				
Right	Medial	Balloon	10 mm depression	No/minimal residual defect
	Lateral	Metal tamp	8 mm depression, comminution	Poor reduction, irregular surface
Left	Medial	Metal tamp	10 mm depression	No/minimal residual defect
	Lateral	Balloon	8 mm depression, comminution	No/minimal residual defect

**Fig. 4:**
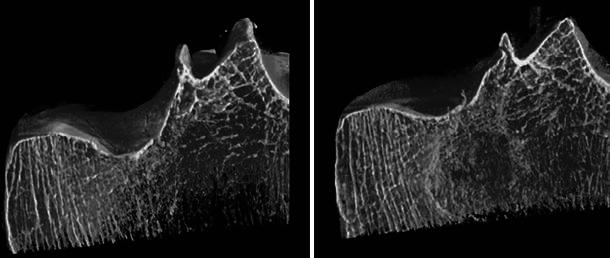
Small depression elevated easily with balloon technique

**Fig. 5:**
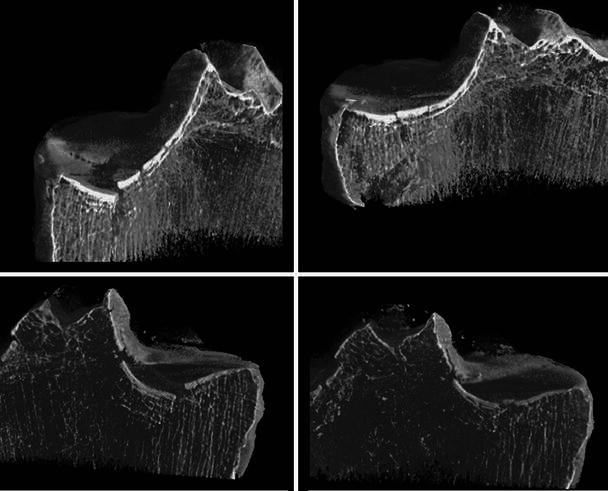
*Top row* shows a depression with large fragments reduced nicely with a metal tamp. *Bottom row* shows similar results when the balloon technique was used

For plateau fractures with a minimal defect (≤4 mm), either technique was capable of a near-perfect reduction (Fig. 4). Likewise, when the depression consisted of one large articular piece, it could be easily elevated with either technique, leaving behind minimal stepoff (Fig. 5).

**Fig. 6:**
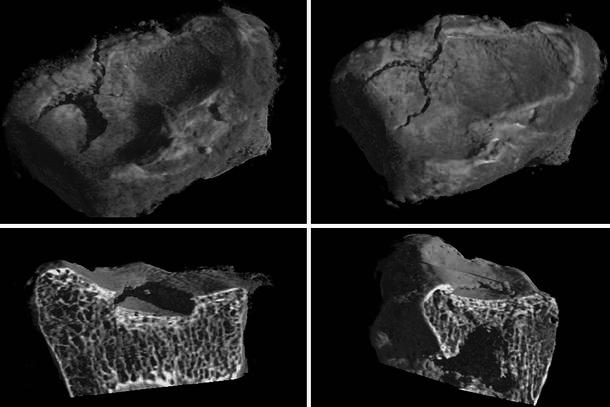
Two examples of die-punch-type distal radius fractures reduced with the balloon

No instances of overreduction or penetration into the joint were encountered with the balloon, whereas this occurred using the metal tamp in three of six tibias. The balloon was equally effective in reducing die-punch fractures of the distal radius (Fig. 6). All five distal radii were reduced with minimal residual defect < 1 mm. The balloon insertion point varied at the distal radius depending on fracture location (styloid, dorsal, or volar). Entry point had no effect on fracture reduction. Again, there were no instances of overreduction or joint-surface penetration.

## Discussion

Anatomic reduction of impacted articular fractures should be the goal of any treating surgeon. In our cadaveric models, we show the inflatable bone tamp to be equivalent to conventional methods (metal tamp or elevator) for reducing depressed intra-articular fractures with large fragments. It was superior to conventional methods for fractures with associated comminution. In addition, no instances of joint penetration were encountered, and over-reduction was not a complication. To our surprise, articular-surface overreduction and penetration was common with minimal force using conventional tamps. It is possible that this is often underappreciated intraoperatively using fluoroscopy.

After inflation and reduction, a defect of known volume was left behind that would be filled with graft or graft substitute. The inflatable bone tamp is less invasive than traditional methods, requiring only a 4- to 5-mm cortical drill hole and only disrupting the underlying cancellous bone where the inflation occurred. Depressed intra-articular fractures can be difficult to treat and acceptable reduction limits vary considerably. Current techniques involve elevating any depressed segments with a metal tamp or elevator and filling the resulting void with bone graft or other desired material [6]. These reduction techniques are often imprecise and have associated complications [1, 2, 14]. With significant comminution, anatomic restoration of the articular surface is virtually impossible to achieve, and conventional instruments can easily penetrate into the joint. Fractures of the articular surface are associated with posttraumatic arthritis and articular cartilage death, and therefore, anatomic restoration of joint congruency should be the goal.

Laboratory studies involving humans and animals reproducibly show that chondrocytes undergo programmed cell death after intra-articular fracture [8, 9, 15]. These models predictably reproduced osteoarthritic changes. Good outcomes were reported with large articular stepoffs as long as joint stability was maintained [6]. One could argue, then, that the goal should be only to restore overall joint alignment and stability with the premise that the articular cartilage is doomed regardless of the degree of reduction. However, if chondrocytes undergo apoptosis after fracture, then some degree of osteoarthritis is going to occur, and worse outcomes have been reported with full-thickness defects and joint congruity loss [7]. If the articular cartilage is to suffer a lethal insult, a congruent reduction is surely preferred over any residual stepoff to minimize wear of the opposing joint surface.

Considering the articular reduction of tibial plateau fractures, much variability remains as to the accepted amount of residual stepoff, with some recommending <3 mm and others accepting up to 10 mm [4, 5]. This is most likely the result of imperfect reduction tools, as anatomic reduction is always preferred. Though posttraumatic arthritis at the knee is correlated more with overall joint alignment and stability, restoring the native anatomy is always the goal. Higher rates of posttraumatic arthritis have been reported after distal radius fractures when >2 mm of step-off remained [16]. Pattee and Thompson [17] recommend reducing the articular surface of the distal radius to within 1 mm to reduce posttraumatic arthritis. Wrist arthroscopy was recommended to better visualize the articular surface and ensure optimal reduction [18].

Altered contact forces have been shown in ankles after intra-articular fractures of the tibial plafond [19]. These forces changed proportionately to the level of residual joint incongruity. The alterations in peak pressure distribution across the articular surface predispose the joint to the development of posttraumatic arthritis [19, 20]. Emphasis is placed on restoring the native articular anatomy to minimize changes in pressure distribution and subsequent arthritic changes.

The inflatable bone tamp appears promising in treating depressed intra-articular fractures of the tibial plateau and distal radius. It appears safe, as no instances of joint penetration, over-reduction, or balloon breakage occurred. In these regions, the balloon appears superior to conventional methods of reducing comminuted intra-articular fracture depressions and equivalent when elevating broad, minimally displaced, fragments. It offers the advantage of being minimally invasive and leaving behind a void of known size and volume. Many isolated joint depression fractures now treated nonoperatively could thus undergo a simple, minimally invasive procedure that would restore articular congruency with minimal morbidity. Caution should be used when generalizing the results of this study due to the small number of specimens used. In addition, we used cadaveric specimens, and the technique will need to be applied to actual patients. We have used the inflatable bone tamp in our trauma center with good success in >20 patients. A clinical study will follow once adequate numbers and follow-up have been achieved.
